# Association between ambient air pollution and daily non-accidental mortality: evidence from the coastal city of Shantou, China

**DOI:** 10.3389/fpubh.2026.1821846

**Published:** 2026-06-18

**Authors:** Yanhu Ji, Yangchun Zheng, Chen Zhou, Xinyi Chen, Zepeng Huang, Guangxing Xu

**Affiliations:** 1 Department of Clinical Medicine, Zhangzhou Health Vocational College, Zhangzhou, Fujian, China; 2 Shantou Center for Disease Control and Prevention, Shantou, Guangdong, China; 3 The Second Affiliated Hospital of Shantou University Medical College, Shantou, Guangdong, China

**Keywords:** air pollution, non-accidental mortality, short-term exposure, susceptible populations, time series study

## Abstract

**Background:**

Current epidemiological evidence on the association between air pollutants and non-accidental mortality remains limited, particularly in developing countries. This study aimed to investigate the short-term association between ambient air pollution and non-accidental mortality, and to quantify the attributable disease burden in Shantou, a subtropical coastal city in China.

**Methods:**

Daily data on non-accidental mortality, meteorological factors, and ambient air pollutants including PM_2.5_, PM_10_, SO_2_, NO_2_, CO, and O_3_ were collected in Shantou from 2016 to 2020. A Poisson generalized additive model (GAM) was applied to estimate the acute effects of air pollutants on non-accidental mortality, with stratification analyses by gender, age, and season. Attributable fractions (AFs) and attributable numbers (ANs) were further estimated based on the World Health Organization (WHO) air quality guidelines and Chinese air quality standards.

**Results:**

Non-accidental mortality in Shantou was significantly associated with exposure to PM_2.5_, PM_10_, SO_2_, NO_2_ and O_3_, but not with CO. Per 10 μg/m^3^ increase in air pollutant concentration, the strongest relative risks (RRs) and 95% confidence intervals (CIs) for non-accidental mortality were 1.0199 (1.0106–1.0294) for PM_2.5_ at lag03 days, 1.0146 (1.0085–1.0209) for PM_10_ at lag 03 days, 1.1268 (1.0773–1.1786) for SO_2_ at lag 07 days, 1.0256 (1.0093–1.0422) for NO_2_ at lag 03 days, and 1.0048 (1.0013–1.0083) for O₃ at lag 04 days, respectively. Stratified analyses showed that individuals aged 65 years and older were more susceptible only to O₃. The significant adverse effect of O₃ was significantly stronger in the warm season than in the cold season, while no significant difference was found between males and females. Overall, 4.23% (5,576 cases) of non-accidental deaths were attributable to excess exposure to air pollution exceeding the WHO air quality guidelines.

**Conclusion:**

Short-term exposure to PM_2.5_, PM_10_, SO_2_, NO_2_ and O_3_ was significantly associated with elevated non-accidental mortality in Shantou, a coastal subtropical city, imposing a considerable disease burden. Our findings highlight the urgent need for effective air pollution control strategies to reduce the related disease burden.

## Introduction

As a prominent global environmental public health issue, air pollution has been confirmed by numerous epidemiological studies as an important risk factor for human health, with short-term or long-term exposure closely associated with an increased risk of non-accidental mortality in the population (1–3). Ambient air pollution, or harmful substances in the surrounding outdoor air, including fine particulate matter (PM₂.₅), inhalable particulate matter (PM₁₀), sulfur dioxide (SO₂), nitrogen dioxide (NO₂) and ozone (O₃), impose adverse effects on human respiratory, cardiovascular and central nervous systems (4–6). These pollutants increase the risks of non-accidental mortality, cognitive impairment, developmental retardation and adverse pregnancy outcomes, indicating an urgent demand for air quality improvement and environmental safety guarantee (7–10). According to the World Health Organization (WHO), nearly 99% of the global population is exposed to air quality levels exceeding recommended limits, and air pollution-related non-accidental deaths have become a key component of the global disease burden, posing a severe challenge to public health security (11).

Shantou, an important port city on the southeast coast of China, features a subtropical marine climate characterized by year-round warmth and humidity. As the economic hub of eastern Guangdong Province, its industrial development, traffic emissions, and residents’ daily activities all generate a certain amount of air pollutants, forming region-specific air pollution patterns. Unlike heavily polluted cities in northern China, Shantou’s air pollution is dominated by ozone and particulate matter; the concentration distribution and seasonal variation of these pollutants are unique, influenced by factors such as marine climate and monsoon circulation (12). Currently, most domestic studies on the association between air pollution and non-accidental mortality have focused on heavily polluted northern cities or economically developed first-tier cities (13–16), while relevant research on subtropical port cities along the southeast coast remains scarce. Specifically, the impact of Shantou’s unique climatic, geographical, and pollution characteristics on population non-accidental mortality has not been clarified, and there is a lack of targeted epidemiological evidence to address this gap.

In view of this, this study took Shantou as the research area and adopted a time-series design to explore the association between short-term exposure to major air pollutants (PM_2.5_, PM_10_, SO_2_, NO_2_, CO and O_3_) and non-accidental mortality in the population. It also analyzed effect modifications by population characteristics (e.g., age and gender) and seasons, and quantified the attributable risk of air pollution-related non-accidental deaths. The results of this study can fill the gap in research on the health effects of air pollution in subtropical port cities along the southeast coast, providing a scientific basis for Shantou to formulate targeted air pollution prevention and control policies, optimize public health interventions, and protect the health of vulnerable populations. Additionally, it offers a valuable reference for air pollution health risk assessment in similar cities across China.

## Materials and methods

### Study area

The study was carried out in Shantou, a coastal city in eastern Guangdong Province bordering the South China Sea. As one of China’s earliest special economic zones, it is also the core birthplace of Chaoshan culture. The city comprises six districts (Chaonan, Chaoyang, Haojiang, Jinping, Longhu, and Chenghai) and Nan’ao County, with a total land area of 2,199 square kilometers. Its terrain declines from the northwest to the southeast, endowed with lengthy coastlines and unique inner bay landforms. Shantou has a permanent population of about 5.58 million with moderate population density, and its regional GDP reached 316.8 billion yuan by the end of 2024. The city boasts solid traditional industries including textiles, garment manufacturing and toy production, while emerging industries such as new energy maintain steady development. It features a southern subtropical maritime monsoon climate with year-round mild and humid weather, abundant rainfall and sufficient sunlight.

### Data collection

The information on date of diagnosis, sex, age, cause of death, as well as non-resident persons and accidental deaths, was retrieved from the Shantou Center for Disease Control and Prevention during the study period from 1 January 2016 to 31 December 2020. Causes of death were classified according to the 10th Revision of the International Classification of Diseases (ICD-10; non-accidental deaths: A00-R99). Non-accidental deaths were subsequently stratified by sex, age, and season.

During the same period, air pollutant data, including daily 24-h average concentrations of PM_2.5_, PM_10_, SO₂, NO₂, CO, and the maximum 8-h average O₃ concentration-were obtained from the China Air Quality Real-time Monitoring Platform^1^. Corresponding meteorological data, including daily average temperature (°C) and relative humidity (%), were extracted from the China Meteorological Science Data Sharing Service Network^2^.

### Statistical analysis

This study employed a time-series design to investigate the association between short-term exposure to air pollutants and non-accidental mortality in Shantou, a coastal city. Descriptive analyses were performed for daily non-accidental deaths, air pollutants (PM_2.5_, PM_10_, SO_2_, NO_2_, O_3_, and CO), and meteorological variables (daily average temperature and relative humidity). Spearman’s correlation analysis was used to examine the collinearity between air pollutants and meteorological variables. The daily count of non-accidental deaths typically follows an overdispersed Poisson distribution; therefore, a generalized additive model (GAM) with a quasi-Poisson link function was applied to explore the relationship between air pollutants and non-accidental mortality. The model specification used in this study is as follows (Equation 1):


$$\begin{array}{l} \log\;E(\mathrm{Yt})=\mathrm{\beta Pt}+\mathrm{ns}(\text{TEMP},3)+\mathrm{ns}\;\;(\mathrm{RH},3)+\text{Holiday}\\ +\mathrm{DOW}+\mathrm{ns}\;\;(\text{time},7\times 5)+\text{intercept} \end{array}$$ (1)

TEMP and RH represent the daily average temperature and relative humidity, respectively; *P*_t_ is the concentration of air pollutant; *t* denotes the observation day and *E*(Y*t*) is the expected number of daily number of non-accidental mortality on day *t*; *β* is the log-relative rate of the exposure-response relationship between air pollutants and non-accidental mortality.

Consistent with previous published studies, the parameters in the model were set as follows:(a) Natural spline (ns) functions with 7 degrees of freedom (*df*) per year were used to control for long-term and seasonal trends (17, 18); (b) Natural spline functions with 3 degrees of freedom per year were applied to adjust for the potential confounding effects of daily average temperature and relative humidity (19, 20); (c) The model was also adjusted for day of week (DOW) effects and holiday effects.

To test the model robustness, we adopted the following strategies. First, single-day lag structures (lag 0 to lag 7) and multi-day moving average lag structures (lag01 to lag07) were used to examine the effects of air pollution on daily non-accidental mortality (21). For instance, lag 0 represented on the day of exposure, while lag01 indicated the moving average exposure on the current and previous days. Second, sensitivity analyses were performed by changing the degrees of freedom for time trends (6–9 *df* per year) and adjusting for other meteorological factors. Third, two-pollutant models were constructed after adjusting for other co-pollutants.

In addition, we conducted subgroup analyses by sex (male and female), age (<65 years old and ≥65 years old), and season (warm season and cold season). Between-group differences were tested using the following formula (Equation 2) (22):


$$(Q1-Q2)\pm \sqrt{(\mathrm{SE}1)2+(\mathrm{SE}2)2}$$ (2)

In the formula, *Q*_1_ and *Q*_2_ are the estimates values of the subgroups, and SE_1_ and SE_2_ represent their standard errors, respectively. Results are expressed as the relative risk (RR) and 95% confidence interval (CI) of daily non-accidental mortality associated with per 10 μg/m^3^ (or 1 mg/m^3^ in CO) increase in pollutant concentrations.

Finally, the disease burden of daily non-accidental mortality attributable to concentrations of air pollutants exceeding the WHO air quality guideline and China air quality primary standard were Calculated. In single-pollutant models, the maximum exposure-response coefficient for daily non-accidental mortality was used to calculate the attributable fraction (AF) and number (AN). The formula is shown below (Equation 3) (21):


$${\text{AN}}_{t}=N_{t}\times [1–\exp.(-\beta )\times ({\text{Pollutant}}_{t}–{\mathrm{WHO}}_{\text{Standard}})]$$ (3)

In Equation 3, *t* denotes day *t*; *N_t_* is the number of daily non-accidental mortality on day *t*; *β* is the exposure-response coefficient derived from Equation 1; *Pollutant_t_ - WHO_Standard_* is the concentration of air pollutant on day *t* minus the reference concentration; and *AN_t_* is the number of daily non-accidental mortality on day *t* attributable to concentrations exceeding the reference value.

All statistical analyses in this study were performed using R software (version 4.0.2) (23), and the generalized additive models were fitted using the “mgcv” package. *p* value < 0.05 was considered statistically significant.

## Results

Table 1 presents the descriptive statistics of daily non-accidental mortality, meteorological factors, and air pollution variables in Shantou from 2016 to 2020. A total of 131,827 non-accidental deaths were recorded during the study period. Of these, 43.9% (57,898) were males and 56.1% (73,929) were females. By age group, the number of non-accidental deaths among those aged 65 years and older (97,800) was 2.87 times that of those under 65 years (34,027). The number of deaths was slightly higher in the cold season than in the warm season (69,503 vs. 62,324). During the study period, the daily average concentrations of PM_2.5_, PM_10_, SO_2_, NO_2_ and CO, as well as the maximum 8-h average concentration of O_3_, were 25.41 μg/m^3^, 43.82 μg/m^3^, 10.82 μg/m^3^, 19.04 μg/m^3^, 0.75 mg/m^3^, and 96.24 μg/m^3^, respectively. The daily average temperature and relative humidity over the same period were 23.63 °C and 76.07%, respectively.

**Table 1:** Description statistics of daily non-accidental mortality, meteorological variables and air pollutants in Shantou, 2016–2020.

Variables	Sum	Mean	SD	Minimum	P10	P25	P50	P75	P90	Maximum
Numbers	131,827	72.15	12.33	38	58	63	71	80	89	121
Males	57,898	31.69	7.27	13	23	27	31	36	41	60
Females	73,929	40.46	7.71	18	31	35	40	46	51	72
<65 years old	34,027	18.62	4.61	5	13	15	18	22	25	34
≥65 years old	97,800	53.53	10.65	27	40	46	53	60	67	96
Warm season	62,324	34.11	34.86	0	0	0	43	67	76	113
Cold season	69,503	38.04	39.15	0	0	0	0	76	86	121
PM_2.5_ (μg/m^3^)	–	25.41	13.13	3	11	16	23	32	44	105
PM_10_ (μg/m^3^)	–	43.82	19.15	7	22	30	40	54	70	159
NO_2_ (μg/m^3^)	–	19.04	7.94	4	11	13	18	23	29	67
SO_2_ (μg/m^3^)	–	10.82	3.67	5	7	8	10	12	16	40
O_3_ (μg/m^3^)	–	96.24	33.94	4	52	68	98	120	141	203
CO (μg/m^3^)	–	746.58	198.94	400	500	600	700	800	1,000	2,700
Mean temperature (°C)	–	23.63	5.65	5.1	15.8	18.9	24.5	28.7	30.3	33.2
Relative humidity (%)	–	76.07	11.25	36.5	61.0	69.0	76.5	84.3	90.0	100.0

PM_2.5_, particulate matter with aerodynamic diameter less than 2.5 μm; PM_10_, particulate matter with aerodynamic diameter less than 10 μm; SO_2_: Sulfur dioxide; NO_2_, nitrogen dioxide; CO, Carbon monoxide; O_3_, Ozone. P_10_, P_25_, P_50_, P_75_, P_90_: the10th, 25th, 50th, 75th, 90th percentile; SD, standard deviation; Cold season, November to April of the next year; Warm season, May to October.

The time series distribution of non-accidental deaths, meteorological factors and air pollutants in Shantou is shown in Supplementary Figure S1. It can be seen that PM_2.5_, PM_10_, SO_2_, NO_2_ and CO all show a decreasing trend year by year, with higher pollutant concentrations in winter and lower in summer. O_3_ levels are higher in summer.

Supplementary Table S1 shows the Spearman correlation coefficients between meteorological factors and air pollutants in Shantou, China. There is a strong positive correlation between PM_2.5_ and PM_10_ (*r_s_* = 0.93, *p* < 0.001), while O_3_ shows weak correlations with SO_2_ (*r_s_* = 0.29, *p* < 0.001), NO_2_ (*r_s_* = 0.21, *p* < 0.001) and CO (*r_s_* = 0.14, *p* < 0.001). Other pollutants exhibit moderate correlations with each other. Daily average temperature and relative humidity are negatively correlated with various pollutants.

A single-pollutant model was used to examine the effects of air pollutants on daily non-accidental mortality. Table 2 shows the relative risks (RR) and 95% confidence intervals (CI) of daily non-accidental mortality associated with each 10 μg/m^3^ increase in air pollutants at specific single-day lags (0–7 days) and moving average lags (lag01–lag07) in the single-pollutant model. We found that short-term exposure to PM_2.5_, PM_10_, SO_2_, NO_2_ and O_3_ was significantly associated with an increased risk of daily non-accidental mortality. At lag03, the relative risks (RR) and 95% confidence intervals (95%CI) of daily non-accidental mortality associated with each 10 μg/m^3^ increase in PM_2.5_, PM_10_, SO_2_, NO_2_ and O_3_ were 1.0199 (1.0106–1.0294), 1.0146 (1.0085–1.0209), 1.0923 (1.0562–1.1295), 1.0256 (1.0093–1.0422), and 1.0037 (1.0004–1.0071), respectively. However, no significant association was observed between CO and daily non-accidental mortality.

**Table 2:** Relative risks of daily non-accidental mortality associated with per 10 μg/m3 increase in air pollutants in single-pollutant models.

Lag	PM_2.5_	PM_10_	SO_2_	NO_2_	CO	O_3_
0	1.0132 (1.0070–1.0196)*	1.0094 (1.0049–1.0139)*	1.0422 (1.0181–1.0670)*	1.0216 (1.0103–1.0330)*	1.0001 (0.9996–1.0005)	1.0006 (0.9979–1.0033)
1	1.0099 (1.0037–1.0162)*	1.0076 (1.0034–1.0120)*	1.0398 (1.0164–1.0637)*	1.0152 (1.0039–1.0266)*	0.9997 (0.9993–1.0001)	1.0008 (0.9984–1.0033)
2	1.0078 (1.0017–1.0141)*	1.0072 (1.0030–1.0114)*	1.0381 (1.0149–1.0619)*	1.0069 (0.9958–1.0181)	0.9995 (0.9991–1.0001)	1.0024 (1.0002–1.0047)*
3	1.0049 (0.9988–1.0111)	1.0048 (1.0006–1.0090)*	1.0473 (1.0239–1.0712)*	1.0054 (0.9943–1.0166)	0.9996 (0.9992–1.0001)	1.0032 (1.0009–1.0054)*
4	1.0008 (0.9948–1.0069)	1.0008 (0.9967–1.0050)	1.0386 (1.0152–1.0625)*	1.0009 (0.9899–1.0121)	0.9998 (0.9994–1.0003)	1.0028 (1.0006–1.0050)*
5	0.9955 (0.9896–1.0015)	0.9986 (0.9945–1.0027)	1.0170 (0.9941–1.0404)	0.9986 (0.9875–1.0098)	0.9999 (0.9995–1.0003)	1.0005 (0.9982–1.0026)
6	0.9965 (0.9906–1.0025)	0.9969 (0.9929–1.0010)	1.0161 (0.9932–1.0395)	1.0018 (0.9906–1.0130)	1.0002 (0.9997–1.0006)	0.9989 (0.9968–1.0011)
7	1.0026 (0.9967–1.0085)	1.0003 (0.9962–1.0043)	1.0129 (0.9900–1.0364)	1.0021 (0.9909–1.0135)	1.0001 (0.9996–1.0005)	0.9992 (0.9970–1.0013)
01	1.0158 (1.0085–1.0232)*	1.0114 (1.0063–1.0166)*	1.0579 (1.0293–1.0873)*	1.0253 (1.0121–1.0387)*	0.9998 (0.9993–1.0004)	1.0010 (0.9981–1.0040)
02	1.0185 (1.0101–1.0269)*	1.0135 (1.0079–1.0193)*	1.0720 (1.0396–1.1053)*	1.0254 (1.0105–1.0405)*	0.9996 (0.9991–1.0002)	1.0023 (0.9992–1.0055)
03	1.0199 (1.0106–1.0294)*	1.0146 (1.0085–1.0209)*	1.0923 (1.0562–1.1295)*	1.0256 (1.0093–1.0422)*	0.9995 (0.9988–1.0001)	1.0037 (1.0004–1.0071)*
04	1.0193 (1.0091–1.0297)*	1.0140 (1.0073–1.0208)*	1.1082 (1.0686–1.1493)*	1.0239 (1.0064–1.0417)*	0.9994 (0.9987–1.0001)	1.0048 (1.0013–1.0083)*
05	1.0161 (1.0051–1.0273)*	1.0126 (1.0054–1.0198)*	1.1132 (1.0704–1.1577)*	1.0217 (1.0032–1.0406)*	0.9994 (0.9987–1.0001)	1.0046 (1.0009–1.0084)*
06	1.0139 (1.0020–1.0256)*	1.0106 (1.0029–1.0183)*	1.1199 (1.0738–1.1680)*	1.0214 (1.0019–1.0414)*	0.9995 (0.9987–1.0003)	1.0038 (0.9999–1.0077)
07	1.0151 (1.0027–1.0278)*	1.0105 (1.0025–1.0186)*	1.1268 (1.0773–1.1786)*	1.0218 (1.0012–1.0429)*	0.9996 (0.9987–1.0004)	1.0033 (0.9992–1.0073)

*p < 0.05.

**Figure 1:**
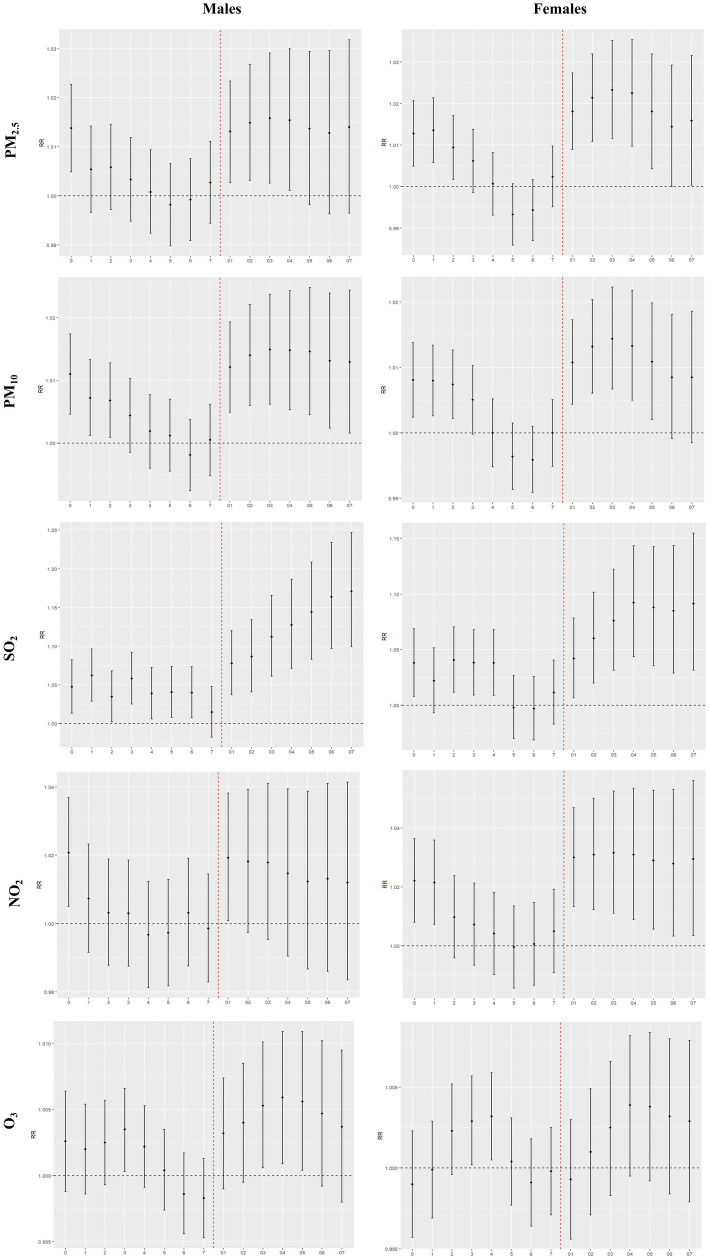
Sex-specific analyses for daily non-accidental mortality associated with 10 µg/m^3^ increase in pollutant concentrations.

**Figure 2:**
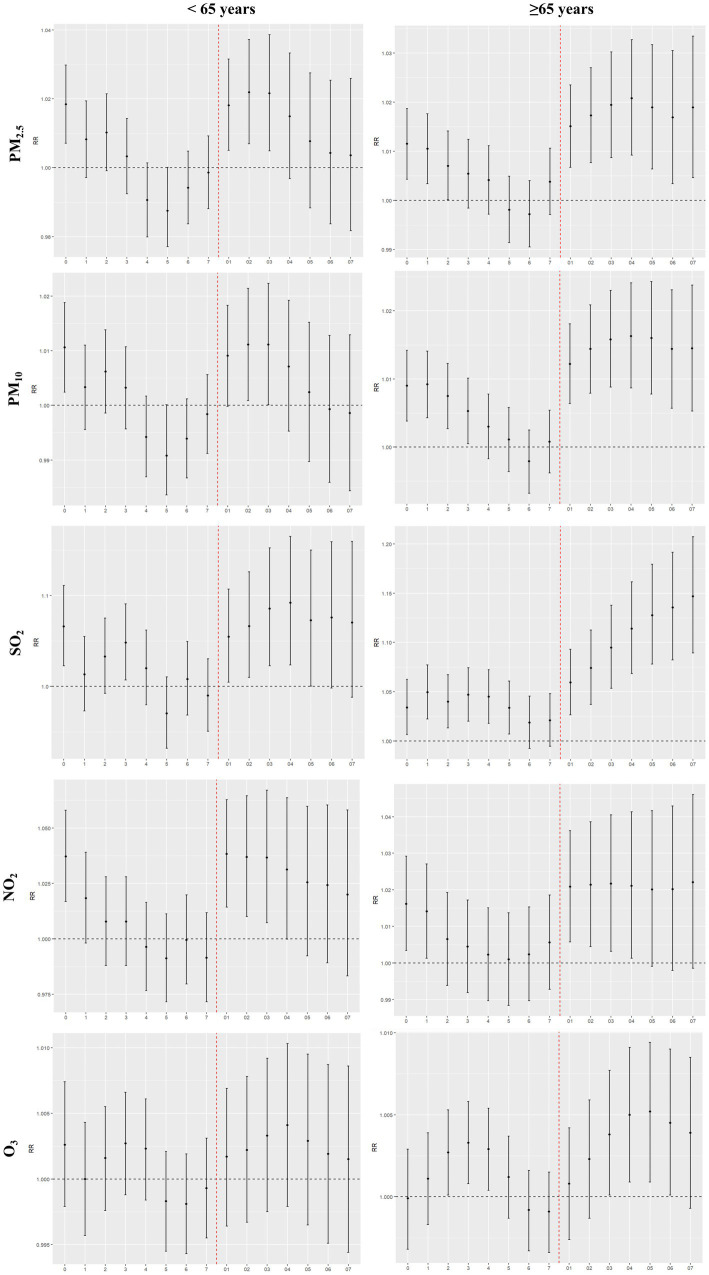
Age-specific analyses for daily non-accidental mortality associated with 10 µg/m^3^ increase in pollutant concentrations.

Figures 1, 2 show the results of sex- and age-specific analyses of daily non-accidental mortality associated with a 10 μg/m^3^ increase in air pollutant concentrations. No significant differences were observed between males and females. Individuals aged 65 years and older appeared to be more susceptible to the effects of O₃ (Supplementary Tables S2–S5). The single-day lag effects of O_3_ on daily non-accidental mortality persisted from lag2 (RR = 1.0027, 95% CI: 1.0001–1.0053) to lag4 (RR = 1.0029, 95% CI: 1.0004–1.0054), and the multi-day lag effects lasted from lag03 (RR = 1.0038, 95% CI: 1.0001–1.0077) to lag06 (RR = 1.0045, 95% CI: 1.0001–1.0090), reaching the maximum at lag05 (RR = 1.0052, 95% CI: 1.0009–1.0094).

**Figure 3:**
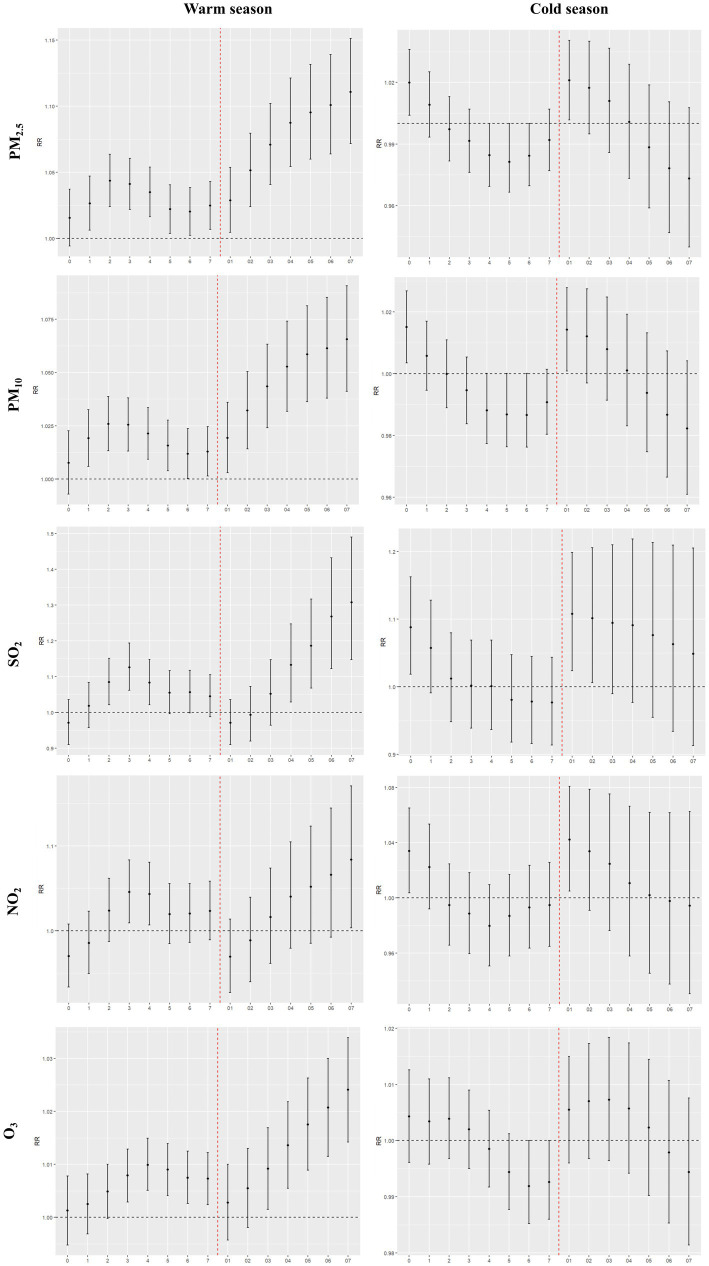
Season-specific analyses for daily non-accidental mortality associated with 10 µg/m^3^ increase in pollutant concentrations.

The results of the season-specific analysis are shown in Figure 3. We found that only the significant adverse effect of O₃ was stronger in the warm season than in the cold season (Supplementary Tables S6, S7). In the warm season group, the single-day lag effects of O_3_ on daily non-accidental mortality persisted from lag 3 (RR = 1.0079, 95% CI: 1.0029–1.0129) to lag 7 (RR = 1.0073, 95% CI: 1.0024–1.0122) and the multi-day lag effects lasted from lag 03 (RR = 1.0092, 95% CI: 1.0015–1.0169) to lag07 (RR = 1.0241, 95% CI: 1.0142–1.0339). However, no significant adverse effects of O₃ were observed in the cold season.

Table 3 shows the attributable fraction (AF) and number (AN) of daily non-accidental mortality due to air pollutants exposure by using different air quality standards. Based on the WHO air quality guidelines, the total proportion of daily non-accidental deaths attributable to excess exposure to air pollutants was 4.23% (5,576 deaths). Specifically, 2.27% (3,004 deaths) were attributable to PM_2.5_, 1.04% (1,372 deaths) to PM_10_, 0.35% (464 deaths) to NO_2_, and 0.56% (736 deaths) to O_3_. Since the daily maximum concentration of SO₂ during the study period did not exceed either the WHO air quality guideline (40 μg/m^3^) or the China air quality primary standard (50 μg/m^3^), the corresponding AN and AF for SO₂ were not calculated.

**Table 3:** Attributable number (AN) and fraction (AF) of daily non-accidental mortality due to air pollutants exposure by using different air quality standards.

Air pollutants	WHO air quality guideline		China air quality primary standard	
	AN (no., 95%CI)	AF (%, 95CI)	AN (no., 95%CI)	AF (%, 95CI)
PM_2.5_	3,004 (2,302, 3,698)	2.27 (1.74, 2.81)	624 (478, 769)	0.47 (0.36, 0.58)
PM_10_	1,372 (1,082, 1,659)	1.04 (0.82, 1.26)	1,029 (811, 1,244)	0.78 (0.61, 0.94)
NO_2_	464 (316, 610)	0.35 (0.24, 0.46)	–	–
O_3_	736 (462, 1,008)	0.56 (0.35, 0.76)	736 (462, 1,008)	0.56 (0.35, 0.76)
Total	5,576 (4,162, 6,975)	4.23 (3.16, 5.29)	2,389 (1751, 3,021)	1.81 (1.32, 2.29)

The strongest effects of air pollutants in single-pollutant models were used (PM_2.5_, lag03 day; PM_10_, lag03 day; SO_2_, lag07 day; NO_2_, lag03 day; O_3_, lag04 day). WHO air quality guidelines: 15 μg/m^3^ for PM_2.5_, 45 μg/m^3^ for PM_10_, 25 μg/m^3^ for NO_2_ and 100 μg/m^3^ for O_3_. China’s primary standards: 35 μg/m^3^ for PM_2.5_, 50 μg/m^3^ for PM_10_, 80 μg/m^3^ for NO_2_ and 100 μg/m^3^ for O_3_. Since the daily maximum concentration of SO_2_ during the study period did not exceed the World Health Organization (WHO) standard (40 μg/m^3^) and the China air quality primary standard (50 μg/m^3^), its attributable numbers (ANs) and attributable fractions (AFs) were not calculated. Similarly, the daily maximum concentration of NO_2_ during the study period (67 μg/m^3^) did not exceed the China air quality primary standard (80 μg/m^3^), so its AFs and ANs were also not calculated.

Supplementary Table S8 shows the relative risks of daily non-accidental mortality associated with each 10 μg/m^3^ increase in air pollutants in the two-pollutant models. The associations remained statistically significant for PM₂·₅, PM₁₀, and SO₂ after adjustment for other air pollutants. In the NO₂ and O₃ models, the effect estimates were attenuated after controlling for particulate matter, while no obvious changes were observed after adjustment for other gaseous pollutants. Furthermore, the effect estimates did not change substantially when varying the degrees of freedom for the time trend (5–9 df) or adjusting for other meteorological factors (rainfall, sunshine duration, atmospheric pressure, and wind speed) in the pollutant models (Supplementary Tables S9, S10).

## Discussion

To date, the association between air pollution and non-accidental causes of death has attracted growing attention. However, evidence from low- and middle-income cities remains limited, particularly in the subtropical regions of southeastern China. Therefore, we conducted a time-series study in Shantou, using Generalized Additive Models (GAMs) to evaluate the impact of ambient air pollutants on non-accidental causes of death, and further estimated the corresponding attributable disease burden. The study results indicate that short-term exposure to PM_2.5_, PM_10_, SO_2_, NO_2_, and O_3_ is significantly associated with an increased risk of non-accidental death. Individuals aged 65 years and older are more susceptible to the adverse effects of O_3_. Moreover, the significant adverse effect of O_3_ is stronger in the warm season. Using the WHO Air Quality Guidelines as the reference standard, 4.23% (5,576 cases) of non-accidental deaths are attributable to excessive exposure to air pollutants.

Air pollutants include a large number of particulate matter and gaseous pollutants. PM_2.5_ has the greatest impact of air pollution on health, which mainly comes from transportation, fuel combustion and industrial processes (24, 25). Long-term exposure to PM_2.5_ can increase all-cause mortality by 8% and cardiovascular events by 10% (26, 27). Studies have also shown that PM_10_ can enter the lungs, and its adverse health outcomes vary depending on the source and composition of the particles (28). In recent years, due to traffic emissions, industrial development, petrochemical activities, arid climatic conditions and sandstorm events, the mortality rate related to air pollution is increasing rapidly (29). This study found that short-term exposure to PM_2.5_ and PM_10_ is significantly associated with non-accidental causes of death in Shantou, which is consistent with the conclusions of some studies at home and abroad. For example, Stafoggia et al. (30). analyzed the impact of PM_10_ on mortality and hospitalization of residents in southern Europe, and found that for every 10 μg/m^3^ increase in PM_10_, the non-accidental mortality rate and hospitalization rate of the population increased by 0.55% (95% CI: 0.24, 0.87%) and 0.65% (95% CI: 0.24, 1.06%), respectively. In a study conducted in China, a study in Jinan (31) found that for every 10 μg/m^3^ increase in PM_10_ and PM_2.5_ concentrations, the non-accidental mortality rate increased by 1.11% (95% CI: 0.96–1.26%) and 0.71% (95% CI: 0.60–0.82%), respectively. Lu et al. (32). summarized and analyzed 59 studies and concluded that for every 10 μg/m^3^ increase in PM_10_, the excess risk of non-accidental mortality, cardiovascular disease mortality and respiratory disease mortality increased by 0.36, 0.36 and 0.42%, respectively. In earlier studies, PM_2.5_ has always been regarded as the most important risk factor for non-accidental death in the population. A meta-analysis by Atkinson et al. (33) pointed out that based on the estimation of 23 all-cause mortality rates, for every 10 μg/m^3^ increase in PM_2.5_, the risk of death increased by 1.04% (95% CI: 0.52 to 1.56%). However, with the increase of control efforts, the impact of PM_2.5_ on total population mortality has shown a downward trend in recent years. When Kuerban et al. (34). compared the impact of PM_2.5_ on population health in 2015 and 2018, they found that in 2018, the number of deaths attributable to PM_2.5_ (total deaths, respiratory disease deaths and cardiovascular disease deaths) and the incidence of chronic bronchitis decreased by 23.4% ~ 26.9% overall. There are several mechanisms underlying the relationship between short-term exposure to particulate pollutants and mortality. For example, particulate matter is a carrier mixed with a variety of toxic components, which can lead to systemic inflammatory response, abnormal changes in blood pressure and plasma viscosity, decreased heart rate variability and arrhythmia (35–37). Compared with other pollutants, particulate pollutants have a strong carrier function and can carry a variety of toxic and harmful substances, including some oxidants, organic compounds and metal elements, which can induce inflammatory responses through reactive oxygen species (38).

Our study also found that short-term exposure to SO₂, NO₂, and O₃ was significantly associated with non-accidental mortality in Shantou, which was consistent with some domestic and international studies. For example, a meta-analysis on NO₂ exposure and mortality reported that each 10 μg/m^3^ increment in NO₂ concentration was associated with increased risks of all-cause, cardiovascular, and respiratory mortality, with pooled RRs of 1.03 (95% CI: 1.02–1.05), 1.07 (95% CI: 1.04–1.10), and 1.03 (95% CI: 1.02–1.05), respectively (39). A survival analysis from South Korea indicated that both NO₂ and SO₂ increased the risk of all-cause mortality, with corresponding effects modified by sex, health behaviors, and socioeconomic status (40). Based on data from 22 European countries between 1973 and 2018, Meng et al. (41) found that each 10 μg/m^3^ increase in NO₂ was associated with 0.46% (95% CI: 0.36–0.57%), 0.37% (95% CI: 0.22–0.51%), and 0.47% (95% CI: 0.21–0.72%) increases in daily all-cause, cardiovascular, and respiratory mortality, respectively. A domestic study covering 17 Chinese cities showed that each 10 μg/m^3^ rise in SO₂ and NO₂ was related to 0.75% (95% CI: 0.45, 1.02) and 1.63% (95% CI: 1.09, 2.17) increases in total mortality, respectively (42). However, Li et al. (43) only observed statistically significant effects of SO₂ on non-accidental mortality when investigating the health impacts of SO₂ and NO₂. In the present study, SO₂ concentrations were consistently low, with daily maximum values below the WHO air quality guidelines. Nevertheless, its multi-day lagged cumulative effects on non-accidental mortality were stronger than those of PM_10_ and PM_2.5_, suggesting that SO₂ exerts greater adverse health impacts regardless of concentration levels and may cause harm as long as it exists. Therefore, stricter measures are still needed to reduce atmospheric SO₂ concentrations as much as possible.

Regarding O₃, domestic studies supported our findings. For instance, in a meta-analysis including 33 time-series studies by Shang et al. (44), only 8 studies focused on O₃; each 10 μg/m^3^ increment in O₃ was associated with a 0.48% increase in total mortality. Furthermore, Chen et al. (45) pooled data from Shanghai, Anshan, and Taiyuan to analyze the short-term associations between CO and daily mortality, and identified significant links of CO with non-accidental and cardiovascular mortality. Each 1 mg/m^3^ rise in CO corresponded to 2.89% (95% CI: 1.68, 4.11) and 4.17% (95% CI: 2.66, 5.68) increases in non-accidental and cardiovascular mortality, respectively. However, no significant adverse effects of CO on non-accidental mortality were observed in this study. The heterogeneous estimates of CO-related mortality risks across studies may be attributable to differences in study populations, regions, and study periods.

Stratified analyses in the present study showed that individuals aged 65 years and older were more susceptible to O₃ exposure, indicating that the older adults are a high-risk group for O₃-related adverse health outcomes. The older adults generally have reduced antioxidant capacity, impaired cardiopulmonary function, and a higher prevalence of chronic comorbidities (46, 47), making them more vulnerable to oxidative stress and inflammatory injury induced by O₃, thus leading to a more significant increase in mortality risk. Meanwhile, stronger effects of O₃ on non-accidental mortality were observed in the warm season. This could be explained by higher temperatures and sufficient sunlight in the warm season, which favor photochemical reactions and result in elevated O₃ concentrations. In addition, people tend to spend more time on outdoor activities in the warm season, leading to prolonged outdoor exposure and further enhancing the health impacts of O₃. These results highlight that priority protection should be given to the older adults when developing health protection strategies against air pollution. Enhanced early warning and intervention measures are warranted during the warm season with severe O₃ pollution to reduce the acute health hazards induced by O₃.

Several limitations of this study should not be ignored. First, the outcome variable of this study was non-accidental mortality among residents, which is a composite cause of death without specific analysis of deaths from particular systemic diseases, and therefore cannot provide targeted prevention for any specific systemic disease. Second, we used city-level average concentrations of air pollutants to represent individual-level exposure, which may lead to exposure misclassification. Third, some potential confounding factors, such as lifestyle, socioeconomic status, and education level, were not included in this study due to data unavailability, and they should be considered in future research. Fourth, as an ecological study, this study is limited in causal inference. Finally, this study is a single-center study, and the results should be generalized with caution to regions with different climatic and geographical characteristics.

## Conclusion

The results indicated that short-term exposure to PM_2.5_, PM_10_, SO_2_, NO_2_, and O_3_ was all significantly associated with an increased risk of daily non-accidental mortality. Individuals aged 65 years and older were more susceptible to O_3_ exposure. The adverse effects of O_3_ were significantly stronger in the warm season than in the cold season, whereas no significant differences were observed between males and females. Additionally, 4.23% (5,576 cases) of non-accidental deaths were attributable to excess exposure to air pollutants beyond the WHO air quality guidelines. This study not only provides epidemiological evidence on the impact of air pollution on daily non-accidental deaths in subtropical cities, but also offers a scientific basis for government departments to formulate targeted air pollution intervention measures.

## Data Availability

The original contributions presented in the study are included in the article/Supplementary material, further inquiries can be directed to the corresponding authors.

## Footnotes

1
https://www.aqistudy.cn/

2
https://data.cma.cn/site/index.html
