# Supplementary material for: Association between ambient air pollution and daily non-accidental mortality: evidence from the coastal city of Shantou, China
Source: Front Public Health. 2026 Jun 18;14:1821846. doi: 10.3389/fpubh.2026.1821846 (PMC13323007; doi:10.3389/fpubh.2026.1821846)
Supplement: Supplementary file 1 [file Data_Sheet_1.doc]

Association between ambient air pollution and daily non-accidental mortality: Evidence from the coastal city of Shantou, China

**Supplementary materials**

**Table S1** Spearman's correlations between air pollutants and meteorological factors

**Fig S1** Time series distributions of daily non-accidental mortality and environmental variables in Shantou, China, 2016-2020

**Table S2** RR and 95% CI of daily non-accidental mortality with a 10 μg/m^3^ increase in concentrations of air pollutants using different lag structures in male group in Shantou, China, 2016-2020

**Table S3** RR and 95% CI of daily non-accidental mortality with a 10 μg/m^3^ increase in concentrations of air pollutants using different lag structures in female group in Shantou, China, 2016-2020

**Table S4** RR and 95% CI of daily non-accidental mortality with a 10 μg/m^3^ increase in concentrations of air pollutants using different lag structures in younger than 65 years group in Shantou, China, 2016-2020

**Table S5** RR and 95% CI of daily non-accidental mortality with a 10 μg/m3 increase in concentrations of air pollutants using different lag structures in older group (≥65 years old ) in Shantou, China, 2016-2020**Table S6** RR and 95% CI of daily non-accidental mortality with a 10 μg/m^3^ increase in concentrations of air pollutants using different lag structures in warm season in Shantou, China, 2016-2020

**Table S7** RR and 95% CI of daily non-accidental mortality with a 10 μg/m^3^ increase in concentrations of air pollutants using different lag structures in cold season in Shantou, China, 2016-2020

**Table S8** Relative risks of daily non-accidental mortality associated with per 10 μg/m3 increase in air pollutants in two-pollutant models

**Table S9** Sensitivity analyses for the smooth function of time trend by using different *dfs* per year

**Table S10** The results of sensitivity analyses after adjusting for other [meteorological](#C:/Users/xiaoh/AppData/Local/youdao/dict/Application/8.9.9.0/resultui/html/index.html) [factor](#C:/Users/xiaoh/AppData/Local/youdao/dict/Application/8.9.9.0/resultui/html/index.html)s (*df*=3)

**Table S1** Spearman's correlations between air pollutants and meteorological factors

|  | PM_10_ | SO_2_ | NO_2_ | CO | O_3_ | Temp | RH |
| --- | --- | --- | --- | --- | --- | --- | --- |
| PM_2.5_ | 0.93*** | 0.65*** | 0.68*** | 0.62*** | 0.51*** | -0.38*** | -0.22*** |
| PM_10_ | - | 0.69*** | 0.65*** | 0.55*** | 0.57*** | -0.30*** | -0.37*** |
| SO_2_ |  | - | 0.65*** | 0.62*** | 0.29*** | -0.15*** | -0.24*** |
| NO_2_ |  |  | - | 0.71*** | 0.21*** | -0.59*** | -0.057* |
| CO |  |  |  | - | 0.14*** | -0.37*** | -0.11*** |
| O_3_ |  |  |  |  | - | -0.14*** | -0.50*** |
| Temp |  |  |  |  |  | - | 0.076** |
| RH |  |  |  |  |  |  | - |

Note. **P*<0.05, ***P*<0.01, ****P*<0.001. Temp: temperature; RH: relative humidity.


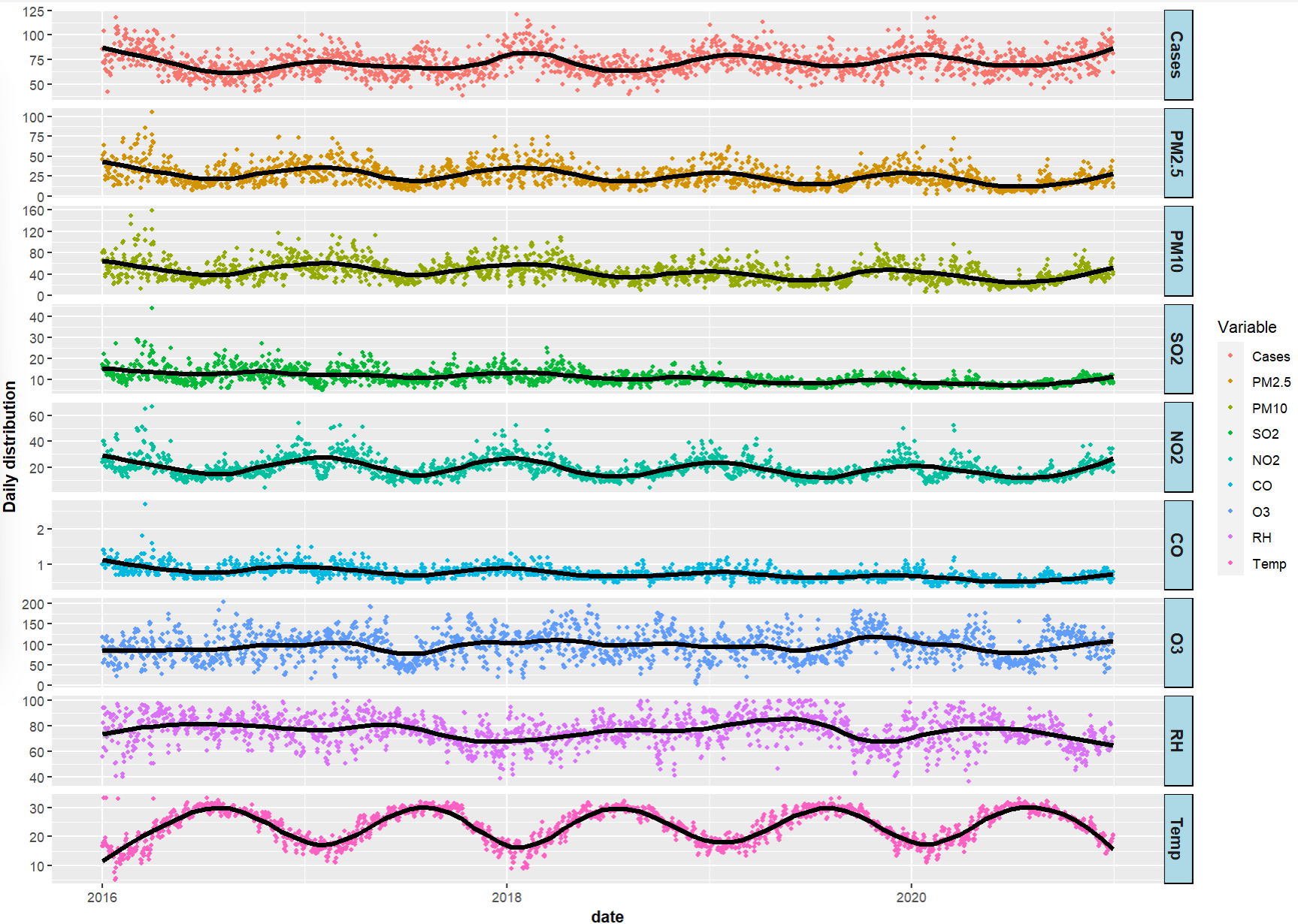


**Fig S1** Time series distributions of daily non-accidental mortality and environmental variables in Shantou, China, 2016-2020. Temp: temperature; RH: relative humidity.

**Table S2** RR and 95% CI of daily non-accidental mortality with a 10 μg/m^3^ increase in concentrations of air pollutants using different lag structures in male group in Shantou, China, 2016-2020

| Lag days | PM_2.5_ | PM_10_ | SO_2_ | NO_2_ | O_3_ |
| --- | --- | --- | --- | --- | --- |
| 0 | 1.0138 (1.0049-1.0227)^*^ | 1.0110 (1.0046-1.0174)^*^ | 1.0473 (1.0133-1.0824)^*^ | 1.0208(1.0050-1.0369)^*^ | 1.0026 (0.9988-1.0064) |
| 1 | 1.0054 (0.9966-1.0142) | 1.0072 (1.0012-1.0133)^*^ | 1.0619 (1.0287-1.0963)^*^ | 1.0073 (0.9916-1.0233) | 1.0020 (0.9986-1.0054) |
| 2 | 1.0058 (0.9972-1.0146) | 1.0068 (1.0009-1.0128)^*^ | 1.0346 (1.0022-1.0680)^*^ | 1.0032 (0.9878-1.0189) | 1.0025 (0.9993-1.0057) |
| 3 | 1.0033 (0.9948-1.0119) | 1.0044 (0.9985-1.0103) | 1.0581 (1.0251-1.0921)^*^ | 1.0030 (0.9875-1.0186) | 1.0035 (1.0003-1.0066)^*^ |
| 4 | 1.0008 (0.9924-1.0094) | 1.0019 (0.9960-1.0077) | 1.0387 (1.0059-1.0725)^*^ | 0.9967 (0.9813-1.0124) | 1.0022 (0.9991-1.0053) |
| 5 | 0.9982 (0.9898-1.0066) | 1.0012 (0.9955-1.0070) | 1.0403 (1.0078-1.0739)^*^ | 0.9973 (0.9818-1.0129) | 1.0004 (0.9974-1.0035) |
| 6 | 0.9992 (0.9909-1.0076) | 0.9981 (0.9924-1.0038) | 1.0399 (1.0073-1.0736)^*^ | 1.0032 (0.9876-1.0191) | 0.9986 (0.9956-1.0017) |
| 7 | 1.0027 (0.9944-1.0111) | 1.0005 (0.9948-1.0062) | 1.0146 (0.9824-1.0478) | 0.9986 (0.9829-1.0145) | 0.9983 (0.9953-1.0013) |
| 01 | 1.0131 (1.0027-1.0234)^*^ | 1.0121 (1.0049-1.0193)^*^ | 1.0776 (1.0371-1.1197)^*^ | 1.0193 (1.0009-1.0382)^*^ | 1.0032 (0.9990-1.0074) |
| 02 | 1.0149 (1.0031-1.0268)^*^ | 1.0140 (1.0060-1.0221)^*^ | 1.0865 (1.0408-1.1342)^*^ | 1.0182 (0.9974-1.0393) | 1.0040 (0.9995-1.0085) |
| 03 | 1.0158 (1.0026-1.0291)^*^ | 1.0149 (1.0062-1.0237)^*^ | 1.1121 (1.0609-1.1657)^*^ | 1.0179 (0.9953-1.0410) | 1.0053 (1.0006-1.0101)^*^ |
| 04 | 1.0154 (1.0011-1.0300)^*^ | 1.0148 (1.0053-1.0243)^*^ | 1.1272 (1.0710-1.1864)^*^ | 1.0147 (0.9905-1.0395) | 1.0059 (1.0009-1.0109)^*^ |
| 05 | 1.0137 (0.9982-1.0294) | 1.0146 (1.0045-1.0248)^*^ | 1.1441 (1.0829-1.2087)^*^ | 1.0123 (0.9867-1.0387) | 1.0056 (1.0004-1.0109)^*^ |
| 06 | 1.0128 (0.9963-1.0296) | 1.0131 (1.0024-1.0239)^*^ | 1.1636 (1.0970-1.2342)^*^ | 1.0131 (0.9860-1.0410) | 1.0047 (0.9992-1.0102) |
| 07 | 1.0140 (0.9965-1.0318) | 1.0129 (1.0016-1.0244)^*^ | 1.1708 (1.0994-1.2469)^*^ | 1.0120 (0.9835-1.0414) | 1.0037 (0.9980-1.0095) |

Note: ^*^*P*<0.05

**Table S3** RR and 95% CI of daily non-accidental mortality with a 10 μg/m^3^ increase in concentrations of air pollutants using different lag structures in female group in Shantou, China, 2016-2020

| Lag days | PM_2.5_ | PM_10_ | SO_2_ | NO_2_ | O_3_ |
| --- | --- | --- | --- | --- | --- |
| 0 | 1.0128 (1.0049-1.0207)^*^ | 1.0081 (1.0024-1.0138)^*^ | 1.0381 (1.0081-1.0690)^*^ | 1.0221 (1.0080-1.0364)^*^ | 0.9990 (0.9957-1.0023) |
| 1 | 1.0136 (1.0058-1.0214)^*^ | 1.0080 (1.0026-1.0134)^*^ | 1.0220 (0.9931-1.0517) | 1.0215 (1.0073-1.0359)^*^ | 0.9999 (0.9969-1.0029) |
| 2 | 1.0094 (1.0017-1.0172)^*^ | 1.0074 (1.0022-1.0127)^*^ | 1.0408 (1.0117-1.0706)^*^ | 1.0098 (0.9959-1.0238) | 1.0023 (0.9996-1.0052) |
| 3 | 1.0062 (0.9986-1.0138) | 1.0051 (0.9998-1.0103) | 1.0383 (1.0093-1.0681)^*^ | 1.0072 (0.9934-1.0213) | 1.0029 (1.0002-1.0057)^*^ |
| 4 | 1.0007 (0.9931-1.0082) | 1.0000 (0.9948-1.0052) | 1.0381 (1.0089-1.0681)^*^ | 1.0042 (0.9903-1.0182) | 1.0032 (1.0005-1.0059)^*^ |
| 5 | 0.9933 (0.9859-1.0007) | 0.9964 (0.9913-1.0015) | 0.9981 (0.9701-1.0270) | 0.9996 (0.9857-1.0136) | 1.0004 (0.9977-1.0031) |
| 6 | 0.9943 (0.9870-1.0017) | 0.9959 (0.9909-1.0010) | 0.9971 (0.9689-1.0259) | 1.0006 (0.9866-1.0147) | 0.9991 (0.9964-1.0018) |
| 7 | 1.0024 (0.9951-1.0098) | 1.0000 (0.9949-1.0051) | 1.0115 (0.9830-1.0408) | 1.0050 (0.9909-1.0192) | 0.9998 (0.9971-1.0025) |
| 01 | 1.0181 (1.0089-1.0274)^*^ | 1.0108 (1.0044-1.0173)^*^ | 1.0421 (1.0068-1.0785)^*^ | 1.0300 (1.0133-1.0469)^*^ | 0.9993 (0.9956-1.0030) |
| 02 | 1.0214 (1.0109-1.0320)^*^ | 1.0132 (1.0061-1.0204)^*^ | 1.0601 (1.0201-1.1017)^*^ | 1.0310 (1.0123-1.0501)^*^ | 1.0010 (0.9971-1.0049) |
| 03 | 1.0233 (1.0116-1.0352)^*^ | 1.0144 (1.0067-1.0223)^*^ | 1.0760 (1.0316-1.1224)^*^ | 1.0316 (1.0111-1.0525)^*^ | 1.0025 (0.9983-1.0066) |
| 04 | 1.0225 (1.0097-1.0354)^*^ | 1.0133 (1.0050-1.0218)^*^ | 1.0924 (1.0436-1.1436)^*^ | 1.0310 (1.0090-1.0535)^*^ | 1.0039 (0.9995-1.0082) |
| 05 | 1.0181 (1.0043-1.0320)^*^ | 1.0109 (1.0020-1.0199)^*^ | 1.0880 (1.0357-1.1429)^*^ | 1.0290 (1.0057-1.0528)^*^ | 1.0038 (0.9992-1.0084) |
| 06 | 1.0144 (1.0000-1.0293)^*^ | 1.0085 (0.9991-1.0181) | 1.0849 (1.0290-1.1438)^*^ | 1.0279 (1.0033-1.0531)^*^ | 1.0032 (0.9984-1.0080) |
| 07 | 1.0159 (1.0003-1.0316)^*^ | 1.0085 (0.9985-1.0186) | 1.0915 (1.0316-1.1548)^*^ | 1.0295 (1.0035-1.0561)^*^ | 1.0029 (0.9979-1.0079) |

Note: ^*^*P*<0.05

**Table S4 RR and 95% CI of daily non-accidental mortality with a 10 μg/m^3^ increase in concentrations of air pollutants using different lag structures in younger than 65 years group in Shantou, China, 2016-2020**

| Lag days | PM_2.5_ | PM_10_ | SO_2_ | NO_2_ | O_3_ |
| --- | --- | --- | --- | --- | --- |
| 0 | 1.0184 (1.0071-1.0298)^*^ | 1.0106 (1.0024-1.0188)^*^ | 1.0660 (1.0228-1.1110)^*^ | 1.0372 (1.0168-1.0580)^*^ | 1.0026 (0.9979-1.0074) |
| 1 | 1.0082 (0.9971-1.0194) | 1.0033 (0.9956-1.0110) | 1.0132 (0.9729-1.0551) | 1.0183 (0.9981-1.0390) | 1.0000 (0.9957-1.0043) |
| 2 | 1.0102 (0.9991-1.0214) | 1.0062 (0.9986-1.0138) | 1.0330 (0.9924-1.0752) | 1.0078 (0.9879-1.0280) | 1.0016 (0.9976-1.0055) |
| 3 | 1.0033 (0.9924-1.0143) | 1.0032 (0.9957-1.0107) | 1.0481 (1.0070-1.0908)^*^ | 1.0078 (0.9880-1.0280) | 1.0027 (0.9988-1.0066) |
| 4 | 0.9906 (0.9799-1.0014) | 0.9942 (0.9869-1.0017) | 1.0199 (0.9796-1.0619) | 0.9964 (0.9766-1.0164) | 1.0023 (0.9984-1.0061)^*^ |
| 5 | 0.9875 (0.9771-1.0001) | 0.9908 (0.9836-1.0000) | 0.9703 (0.9318-1.0103) | 0.9912 (0.9716-1.0113) | 0.9983 (0.9945-1.0021) |
| 6 | 0.9942 (0.9837-1.0048) | 0.9939 (0.9867-1.0012) | 1.0080 (0.9683-1.0493) | 0.9995 (0.9797-1.0198) | 0.9981 (0.9943-1.0019) |
| 7 | 0.9986 (0.9881-1.0092) | 0.9984 (0.9912-1.0056) | 0.9898 (0.9506-1.0305) | 0.9915 (0.9716-1.0118) | 0.9993 (0.9955-1.0031) |
| 01 | 1.0181 (1.0050-1.0315)^*^ | 1.0091 (0.9998-1.0183) | 1.0546 (1.0046-1.1071)^*^ | 1.0383 (1.0143-1.0628)^*^ | 1.0017 (0.9964-1.0069) |
| 02 | 1.0219 (1.0069-1.0372)^*^ | 1.0111 (1.0009-1.0214)^*^ | 1.0663 (1.0098-1.1261)^*^ | 1.0369 (1.0100-1.0645)^*^ | 1.0022 (0.9967-1.0078) |
| 03 | 1.0216 (1.0049-1.0386)^*^ | 1.0111 (1.0000-1.0223)^*^ | 1.0857 (1.0228-1.1525)^*^ | 1.0367 (1.0073-1.0670)^*^ | 1.0033 (0.9975-1.0092) |
| 04 | 1.0149 (0.9968-1.0333) | 1.0071 (0.9953-1.0192) | 1.0920 (1.0235-1.1650)^*^ | 1.0313 (0.9999-1.0637) | 1.0041 (0.9979-1.0103) |
| 05 | 1.0077 (0.9883-1.0275) | 1.0024 (0.9897-1.0152) | 1.0727 (1.0004-1.1502)^*^ | 1.0255 (0.9924-1.0598) | 1.0029 (0.9965-1.0095) |
| 06 | 1.0043 (0.9837-1.0254) | 0.9993 (0.9859-1.0128) | 1.0757 (0.9981-1.1594) | 1.0242 (0.9892-1.0604) | 1.0019 (0.9951-1.0087) |
| 07 | 1.0036 (0.9817-1.0259) | 0.9986 (0.9844-1.0129) | 1.0703 (0.9880-1.1595) | 1.0200 (0.9833-1.0581) | 1.0015 (0.9944-1.0086) |

Note: ^*^*P*<0.05

**Table S5** RR and 95% CI of daily non-accidental mortality with a 10 μg/m^3^ increase in concentrations of air pollutants using different lag structures in older group (≥65 years old ) in Shantou, China, 2016-2020

| Lag days | PM_2.5_ | PM_10_ | SO_2_ | NO_2_ | O_3_ |
| --- | --- | --- | --- | --- | --- |
| 0 | 1.0115 (1.0043-1.0187)^*^ | 1.0090 (1.0038-1.0142)^*^ | 1.0341 (1.0066-1.0624)^*^ | 1.0162 (1.0034-1.0292)^*^ | 0.9999 (0.9968-1.0029) |
| 1 | 1.0105 (1.0034-1.0176)^*^ | 1.0092 (1.0043-1.0141)^*^ | 1.0495 (1.0225-1.0771)^*^ | 1.0141 (1.0013-1.0271)^*^ | 1.0011 (0.9983-1.0039) |
| 2 | 1.0070 (1.0000-1.0141)^*^ | 1.0075 (1.0027-1.0123)^*^ | 1.0400 (1.0134-1.0674)^*^ | 1.0065 (0.9939-1.0193) | 1.0027 (1.0001-1.0053)^*^ |
| 3 | 1.0054 (0.9984-1.0124) | 1.0053 (1.0005-1.0101)^*^ | 1.0469 (1.0202-1.0743)^*^ | 1.0045 (0.9919-1.0172) | 1.0033 (1.0008-1.0058)^*^ |
| 4 | 1.0041 (0.9972-1.0111) | 1.0030 (0.9983-1.0078) | 1.0449 (1.0180-1.0724)^*^ | 1.0023 (0.9897-1.0151) | 1.0029 (1.0004-1.0054)^*^ |
| 5 | 0.9981 (0.9914-1.0049) | 1.0011 (0.9964-1.0058) | 1.0336 (1.0071-1.0608)^*^ | 1.0010 (0.9884-1.0137) | 1.0012 (0.9987-1.0037) |
| 6 | 0.9972 (0.9905-1.0040) | 0.9979 (0.9932-1.0025) | 1.0187 (0.9924-1.0457) | 1.0024 (0.9897-1.0153) | 0.9992 (0.9967-1.0016) |
| 7 | 1.0038 (0.9971-1.0106) | 1.0008 (0.9962-1.0054) | 1.0210 (0.9946-1.0481) | 1.0056 (0.9928-1.0186) | 0.9991 (0.9966-1.0015) |
| 01 | 1.0151 (1.0067-1.0235)^*^ | 1.0122 (1.0064-1.0181)^*^ | 1.0593 (1.0266-1.0931)^*^ | 1.0209 (1.0058-1.0362)^*^ | 1.0008 (0.9974-1.0042) |
| 02 | 1.0173 (1.0077-1.0270)^*^ | 1.0144 (1.0079-1.0209)^*^ | 1.0742 (1.0372-1.1126)^*^ | 1.0214 (1.0045-1.0386)^*^ | 1.0023 (0.9987-1.0059) |
| 03 | 1.0194 (1.0087-1.0302)^*^ | 1.0158 (1.0088-1.0230)^*^ | 1.0947 (1.0534-1.1376)^*^ | 1.0217 (1.0032-1.0405)^*^ | 1.0038 (1.0001-1.0077)^*^ |
| 04 | 1.0208 (1.0092-1.0327)^*^ | 1.0163 (1.0087-1.0241)^*^ | 1.1140 (1.0685-1.1614)^*^ | 1.0211 (1.0013-1.0413)^*^ | 1.0050 (1.0009-1.0091)^*^ |
| 05 | 1.0189 (1.0064-1.0317)^*^ | 1.0160 (1.0078-1.0243)^*^ | 1.1275 (1.0781-1.1792)^*^ | 1.0201 (0.9991-1.0416) | 1.0052 (1.0009-1.0094)^*^ |
| 06 | 1.0169 (1.0034-1.0305)^*^ | 1.0144 (1.0057-1.0231)^*^ | 1.1355 (1.0822-1.1914)^*^ | 1.0202 (0.9980-1.0429) | 1.0045 (1.0001-1.0090)^*^ |
| 07 | 1.0189 (1.0046-1.0334)^*^ | 1.0145 (1.0053-1.0238)^*^ | 1.1468 (1.0894-1.2071)^*^ | 1.0221 (0.9986-1.0460) | 1.0039 (0.9993-1.0085) |

Note: ^*^*P*<0.05

**Table S6** RR and 95% CI of daily non-accidental mortality with a 10 μg/m^3^ increase in concentrations of air pollutants using different lag structures in warm season in Shantou, China, 2016-2020

| Lag days | PM_2.5_ | PM_10_ | SO_2_ | NO_2_ | O_3_ |
| --- | --- | --- | --- | --- | --- |
| 0 | 1.0155 (0.9942-1.0372) | 1.0076 (0.9929-1.0226) | 0.9708 (0.9097-1.0359)^*^ | 0.9701 (0.9337-1.0078) | 1.0013 (0.9948-1.0078) |
| 1 | 1.0265 (1.0062-1.0472)^*^ | 1.0191 (1.0059-1.0325)^*^ | 1.0184 (0.9572-1.0836) | 0.9854 (0.9492-1.0230) | 1.0025 (0.9969-1.0082) |
| 2 | 1.0436 (1.0239-1.0636)^*^ | 1.0259 (1.0132-1.0387)^*^ | 1.0845 (1.0217-1.1512)^*^ | 1.0237 (0.9871-1.0616) | 1.0049 (0.9998-1.0100) |
| 3 | 1.0411 (1.0219-1.0605)^*^ | 1.0255 (1.0131-1.0381)^*^ | 1.1260 (1.0620-1.1938)^*^ | 1.0455 (1.0090-1.0834)^*^ | 1.0079 (1.0029-1.0129)^*^ |
| 4 | 1.0350 (1.0163-1.0540)^*^ | 1.0213 (1.0092-1.0335)^*^ | 1.0828 (1.0216-1.1477)^*^ | 1.0431 (1.0068-1.0806)^*^ | 1.0099 (1.0051-1.0149)^*^ |
| 5 | 1.0221 (1.0038-1.0406)^*^ | 1.0157 (1.0039-1.0277)^*^ | 1.0551 (0.9964-1.1171) | 1.0196 (0.9848-1.0557) | 1.0090 (1.0041-1.0139)^*^ |
| 6 | 1.0202 (1.0022-1.0384)^*^ | 1.0118 (1.0002-1.0236)^*^ | 1.0562 (0.9981-1.1176) | 1.0203 (0.9860-1.0557) | 1.0075 (1.0026-1.0125)^*^ |
| 7 | 1.0248 (1.0069-1.0430)^*^ | 1.0129 (1.0014-1.0246)^*^ | 1.0449 (0.9877-1.1054) | 1.0232 (0.9892-1.0584) | 1.0073 (1.0024-1.0122)^*^ |
| 01 | 1.0288 (1.0045-1.0537)^*^ | 1.0193 (1.0029-1.0360)^*^ | 0.9708 (0.9097-1.0359) | 0.9693 (0.9271-1.0135) | 1.0028 (0.9957-1.0100) |
| 02 | 1.0514 (1.0241-1.0795)^*^ | 1.0321 (1.0142-1.0504)^*^ | 0.9933 (0.9199-1.0725) | 0.9884 (0.9399-1.0394) | 1.0055 (0.9981-1.0130) |
| 03 | 1.0710 (1.0408-1.1021)^*^ | 1.0435 (1.0241-1.0633)^*^ | 1.0519 (0.9644-1.1472) | 1.0160 (0.9613-1.0737) | 1.0092 (1.0015-1.0169)^*^ |
| 04 | 1.0874 (1.0544-1.1214)^*^ | 1.0527 (1.0318-1.0741)^*^ | 1.1331 (1.0293-1.2473)^*^ | 1.0402 (0.9793-1.1048) | 1.0136 (1.0055-1.0218)^*^ |
| 05 | 1.0953 (1.0600-1.1317)^*^ | 1.0585 (1.0362-1.0813)^*^ | 1.1860 (1.0680-1.3170)^*^ | 1.0519 (0.9850-1.1234) | 1.0175 (1.0089-1.0263)^*^ |
| 06 | 1.1009 (1.0639-1.1392)^*^ | 1.0613 (1.0379-1.0852)^*^ | 1.2677 (1.1221-1.4323)^*^ | 1.0657 (0.9923-1.1445) | 1.0207 (1.0115-1.0300)^*^ |
| 07 | 1.1107 (1.0717-1.1512)^*^ | 1.0656 (1.0411-1.0906)^*^ | 1.3073 (1.1472-1.4898)^*^ | 1.0837 (1.0032-1.1707)^*^ | 1.0241 (1.0142-1.0339)^*^ |

Note: ^*^*P*<0.05

**Table S7** RR and 95% CI of daily non-accidental mortality with a 10 μg/m^3^ increase in concentrations of air pollutants using different lag structures in cold season in Shantou, China, 2016-2020

| Lag days | PM_2.5_ | PM_10_ | SO_2_ | NO_2_ | O_3_ |
| --- | --- | --- | --- | --- | --- |
| 0 | 1.0199 (1.0040-1.0361)^*^ | 1.0151 (1.0035-1.0268)^*^ | 1.0882 (1.0187-1.1625)^*^ | 1.0339 (1.0036-1.0650)^*^ | 1.0043 (0.9961-1.0126) |
| 1 | 1.0091 (0.9933-1.0252) | 1.0057 (0.9946-1.0170) | 1.0575 (0.9911-1.1283) | 1.0222 (0.9919-1.0535) | 1.0034 (0.9958-1.0110) |
| 2 | 0.9972 (0.9817-1.0131) | 0.9999 (0.9890-1.0109) | 1.0121 (0.9484-1.0800) | 0.9947 (0.9657-1.0245) | 1.0039 (0.9968-1.0112) |
| 3 | 0.9915 (0.9762-1.0070) | 0.9946 (0.9838-1.0054) | 1.0018 (0.9388-1.0689) | 0.9885 (0.9595-1.0183) | 1.0020 (0.9950-1.0090) |
| 4 | 0.9845 (0.9694-1.0001) | 0.9881 (0.9774-1.0001) | 1.0008 (0.9369-1.0691) | 0.9797 (0.9506-1.0096) | 0.9985 (0.9917-1.0054) |
| 5 | 0.9813 (0.9665-1.0000) | 0.9868 (0.9763-1.0000) | 0.9807 (0.9182-1.0474) | 0.9869 (0.9578-1.0169) | 0.9944 (0.9877-1.0012) |
| 6 | 0.9843 (0.9696-1.0001) | 0.9866 (0.9762-1.0000) | 0.9783 (0.9159-1.0450) | 0.9931 (0.9635-1.0235) | 0.9919 (0.9852-1.0000) |
| 7 | 0.9919 (0.9770-1.0070) | 0.9908 (0.9804-1.0014) | 0.9766 (0.9138-1.0438) | 0.9948 (0.9648-1.0256) | 0.9926 (0.9860-1.0000) |
| 01 | 1.0210 (1.0018-1.0405)^*^ | 1.0142 (1.0008-1.0278)^*^ | 1.1078 (1.0236-1.1989)^*^ | 1.0421 (1.0048-1.0808)^*^ | 1.0055 (0.9960-1.0150) |
| 02 | 1.0173 (0.9949-1.0401) | 1.0121 (0.9970-1.0274) | 1.1014 (1.0062-1.2057)^*^ | 1.0338 (0.9908-1.0787) | 1.0070 (0.9968-1.0173) |
| 03 | 1.0110 (0.9859-1.0367) | 1.0079 (0.9914-1.0248) | 1.0944 (0.9896-1.2102) | 1.0246 (0.9763-1.0753) | 1.0073 (0.9964-1.0184) |
| 04 | 1.0007 (0.9732-1.0289) | 1.0010 (0.9831-1.0192) | 1.0910 (0.9766-1.2187) | 1.0106 (0.9577-1.0664) | 1.0057 (0.9942-1.0174) |
| 05 | 0.9884 (0.9589-1.0188) | 0.9938 (0.9747-1.0132) | 1.0764 (0.9549-1.2134) | 1.0019 (0.9453-1.0620) | 1.0023 (0.9902-1.0145) |
| 06 | 0.9782 (0.9468-1.0106) | 0.9867 (0.9665-1.0073) | 1.0630 (0.9340-1.2097) | 0.9976 (0.9375-1.0616) | 0.9979 (0.9853-1.0107) |
| 07 | 0.9732 (0.9399-1.0077) | 0.9823 (0.9610-1.0041) | 1.0490 (0.9131-1.2051) | 0.9943 (0.9305-1.0625) | 0.9944 (0.9814-1.0076) |

Note: ^*^*P*<0.05

**Table S8** Relative risks of daily non-accidental mortality associated with per 10 μg/m^3^ increase in air pollutants in two-pollutant models

| Model | RR | 95% CI |
| --- | --- | --- |
| PM_2.5_ |  |  |
| Single pollutant model | 1.0199 | 1.0106-1.0294* |
| +SO_2_ | 1.0168 | 1.0070-1.0265* |
| +NO_2_ | 1.0167 | 1.0071-1.0266* |
| +CO | 1.0219 | 1.0121-1.0318* |
| +O_3_ | 1.0209 | 1.0112-1.0308* |
| PM_10_ |  |  |
| Single pollutant model | 1.0146 | 1.0085-1.0209* |
| +SO_2_ | 1.0126 | 1.0061-1.0191* |
| +NO_2_ | 1.0123 | 1.0058-1.0188* |
| +CO | 1.0154 | 1.0090-1.0218* |
| +O_3_ | 1.0151 | 1.0087-1.0216* |
| NO_2_ |  |  |
| Single pollutant model | 1.0256 | 1.0093-1.0422* |
| +PM_2.5_ | 1.0169 | 0.9995-1.0347 |
| +PM_10_ | 1.0160 | 0.9987-1.0336 |
| +SO_2_ | 1.0158 | 1.0001-1.0343* |
| +CO | 1.0299 | 1.0116-1.0485* |
| +O_3_ | 1.0259 | 1.0094-1.0428* |
| SO_2_ |  |  |
| Single pollutant model | 1.1268 | 1.0773-1.1786* |
| +PM_2.5_ | 1.1152 | 1.0645-1.1683* |
| +PM_10_ | 1.1131 | 1.0624-1.1662* |
| +NO_2_ | 1.1042 | 1.0525-1.1584* |
| +CO | 1.1297 | 1.0793-1.1825* |
| +O_3_ | 1.1270 | 1.0772-1.1789* |
| O_3_ |  |  |
| Single pollutant model | 1.0048 | 1.0013-1.0083* |
| +PM_2.5_ | 1.0021 | 0.9983-1.0059 |
| +PM_10_ | 1.0024 | 0.9987-1.0061 |
| +SO_2_ | 1.0040 | 1.0005-1.0076* |
| +CO | 1.0053 | 1.0018-1.0089* |
| +NO_2_ | 1.0046 | 1.0011-1.0082* |

**Note:** **P* < 0.05.

The strongest effects of air pollutants in single-pollutant models were used (PM_2.5_, lag03 day; PM_10_, lag03 day; SO_2_, lag07 day; NO_2_, lag03 day; O_3_, lag04 day)

**Table S9** Sensitivity analyses for the smooth function of time trend by using different *dfs* per year

| *df* | PM_2.5_ | PM_10_ | SO_2_ | NO_2_ | O_3_ |
| --- | --- | --- | --- | --- | --- |
| 5 | 1.0191 (1.0099-1.0283)* | 1.0143 (1.0083-1.0204)* | 1.0742 (1.0340-1.1159)* | 1.0215 (1.0068-1.0363)* | 1.0031 (1.0001-1.0065)* |
| 6 | 1.0221 (1.0127-1.0315)* | 1.0165 (1.0104-1.0227)* | 1.1186 (1.0742-1.1647)* | 1.0297 (1.0145-1.0452)* | 1.0036 (1.0001-1.0072)* |
| 7 | 1.0199 (1.0106-1.0294)* | 1.0146 (1.0085-1.0209)* | 1.1268 (1.0773-1.1786)* | 1.0256 (1.0093-1.0422)* | 1.0048 (1.0013-1.0083)* |
| 8 | 1.0198 (1.0103-1.0294)* | 1.0143 (1.0080-1.0206)* | 1.1449 (1.0917-1.2007)* | 1.0296 (1.0121-1.0474)* | 1.0046 (1.0010-1.0082)* |
| 9 | 1.0211 (1.0115-1.0308)* | 1.0142 (1.0078-1.0205)* | 1.1586 (1.1035-1.2165)* | 1.0366 (1.0182-1.0553)* | 1.0043 (1.0008-1.0079)* |

**Note:** **P* < 0.05. The strongest effects of air pollutants in single-pollutant models were used (PM_2.5_, lag03 day; PM_10_, lag03 day; SO_2_, lag07 day; NO_2_, lag03 day; O_3_, lag04 day)

**Table S10** The results of sensitivity analyses after adjusting for other [meteorological](#C:/Users/xiaoh/AppData/Local/youdao/dict/Application/8.9.9.0/resultui/html/index.html) [factor](#C:/Users/xiaoh/AppData/Local/youdao/dict/Application/8.9.9.0/resultui/html/index.html)s (*df*=3)

| **Factors** | Model used in the article^＃^ | + Rainfall | + Sunshine duration | +Atmospheric pressure | + Wind speed |
| --- | --- | --- | --- | --- | --- |
| PM_2.5_ | 1.0199 (1.0106-1.0294)* | 1.0201 (1.0107-1.0296)* | 1.0202 (1.0108-1.0296)* | 1.0200 (1.0106-1.0295)* | 1.0194 (1.0101-1.0288)* |
| PM_10_ | 1.0146 (1.0085-1.0209)* | 1.0147 (1.0085-1.0209)* | 1.0148 (1.0086-1.0210)* | 1.0146 (1.0084-1.0209)* | 1.0143 (1.0081-1.0206)* |
| SO_2_ | 1.1268 (1.0773-1.1786)* | 1.1277 (1.0781-1.1795)* | 1.1257 (1.0762-1.1774)* | 1.1293 (1.0796-1.1813)* | 1.1251 (1.0757-1.1767)* |
| NO_2_ | 1.0256 (1.0093-1.0422)* | 1.0266 (1.0102-1.0432)* | 1.0261 (1.0098-1.0427)* | 1.0266 (1.0102-1.0432)* | 1.0242 (1.0078-1.0409)* |
| O_3_ | 1.0048 (1.0013-1.0083)* | 1.0047 (1.0012-1.0083)* | 1.0045 (1.0009-1.0080)* | 1.0047 (1.0012-1.0083)* | 1.0049 (1.0014-1.0084)* |

^＃^The model covariates include long-term and seasonal trends, average temperature, relative humidity, day of the week and holiday effects.

**P*<0.05; PM_2.5_, lag03 day; PM_10_, lag03 day; SO_2_, lag07 day; NO_2_, lag03 day; O_3_, lag04 day
